# Differential utility of teacher and parent–teacher combined information in the assessment of Attention Deficit/Hyperactivity Disorder symptoms

**DOI:** 10.1007/s00787-020-01509-4

**Published:** 2020-04-03

**Authors:** Alexandra Garcia-Rosales, Silia Vitoratou, Stephen V. Faraone, Daniel Rudaizky, Tobias Banaschewski, Philip Asherson, Edmund Sonuga-Barke, Jan Buitelaar, Robert D. Oades, Aribert Rothenberger, Hans-Christoph Steinhausen, Eric Taylor, Wai Chen

**Affiliations:** 1 MRC Social Genetic Developmental and Psychiatry Centre, Institute of Psychiatry, Psychology, and Neurosciences, King’s College London, London, UK; 2 Psychometrics and Measurement Lab, Department of Biostatistics and Health Informatics, Institute of Psychiatry, Psychology, and Neurosciences, King’s College London, London, UK; 3 Universidad Autónoma de Madrid, Madrid, Spain; 4 Departments of Psychiatry and of Neuroscience and Physiology, SUNY Upstate Medical University, Syracuse, NY USA; 5 Centre for the Advancement of Research on Emotion, School of Psychological Sciences, University of Western Australia, Perth, Australia; 6 Department of Child and Adolescent Psychiatry and Psychotherapy, Central Institute of Mental Health, Medical Faculty Mannheim/Heidelberg University, Mannheim, Germany; 7 Department of Cognitive Neuroscience, Donders Institute for Brain, Cognition and Behaviour, Radboud University Medical Center, Nijmegen, The Netherlands; 8 Clinic for Child and Adolescent Psychiatry and Psychotherapy, University of Duisburg-Essen, Essen, Germany; 9 Clinic for Child and Adolescent Psychiatry and Psychotherapy, University Medical Center, Göttingen, Germany; 10 Department of Child and Adolescent Psychiatry, University of Zurich, Zurich, Switzerland; 11 Clinical Psychology and Epidemiology, Department of Psychology, University of Basel, Basel, Switzerland; 12 Child and Adolescent Mental Health Center, Capital Region Psychiatry, Copenhagen, Denmark; 13 Department of Child and Adolescent Psychiatry, Southern Denmark University, Odense, Denmark; 14 Complex Attention and Hyperactivity Disorders Service (CAHDS), Child and Adolescent Health Service (CAHS), Department of Health Western Australia, Crawley, Australia; 15 Faculty of Health and Medical Sciences, Paediatrics, University of Western Australia, Crawley, Australia; 16 Centre for Child & Adolescent Related Disorders, Graduate School of Education, University of Western Australia, Crawley, Australia

**Keywords:** ADHD DSM-IV criteria, Caseness, Parent/teacher, IRT, Diagnostic overshadowing

## Abstract

**Background:**

Consistent research findings indicate that parents and teachers observe genuinely different Attention Deficit/Hyperactivity Disorder (ADHD) behaviours in their respective settings.

**Objective:**

To evaluate the utility of information provided by teacher informant assessments (INFAs) of ADHD symptoms, and the implications of aggregation algorithms in combing parents’ information, i.e. using ‘or-rule’ (endorsement by either one informant) versus ‘and-rule’ (endorsement by both informants).

**Method:**

Teacher ratings on Conners scales and clinical data from parental accounts on 1383 probands and their siblings from the IMAGE study were analysed. The psychometric properties of teacher and combined ratings using the item response theory model (IRT) are presented. Kappa coefficients, intraclass correlations and linear regression were employed.

**Results:**

First, teacher endorsement of symptoms is located in a narrow part of the trait continuum close to the average levels. Symptoms exhibit comparable perception in the measurement of the trait(s) with similar discrimination ability and information (reliability). Second, the IRT properties of the ‘or-rule’ ratings are predominantly influenced by parent-INFAs; and the ‘and-rule’ ratings predominantly by teacher-INFAs ratings. Third, parent-teacher INFAs agreement was low, both for individual items (*κ* = 0.01–0.15) and for dimensional scores (*r* = 0.12–0.16). The ‘or-rule’ captured milder expressions of ADHD symptoms, whereas the ‘and-rule’ indexed greater severity of ADHD.

**Conclusions:**

Parent and teacher-INFAs provide different kinds of information, while both are useful. Teacher-INFA and the ‘and-rule’ provide a more accurate index of severity than an additive symptom count. Parent-INFA and the ‘or-rule’ are more sensitive for detecting cases with milder ADHD.

**Electronic supplementary material:**

The online version of this article (10.1007/s00787-020-01509-4) contains supplementary material, which is available to authorized users.

## Introduction

Attention Deficit/Hyperactivity Disorder (ADHD) is one of the most frequently diagnosed child psychiatric disorders in clinical practice, affecting about 3–5% of all school age children. Authoritative diagnostic manuals and practice guidelines recommend aggregating information for diagnostic purposes across informants and settings, in particular, from the home and school settings, with information typically derived from parents and teachers [1].

However, the agreement between parent and teacher ratings of ADHD symptoms is typically low to moderate with different studies reporting correlations between these ratings ranging from 0.09 to 0.43 [2]

Tripp et al. [3] found that parent ratings were similar for children with or without ADHD, but teachers outperformed parents in terms of diagnostic discrimination. Hartman et al. [4] reported that teachers display less bias in ADHD ratings.

Other authors have previously carried out item-response theory (IRT) analyses on parent and teacher ratings using different instruments. Briefly, Gomez [5] carried out IRT analyses on both parent and teacher ratings using the DARS (DSM-IV ADHD rating scale). Gomez et al. [6] as well as Arias et al. [7] examined teacher ratings and found that symptoms represented traits located from mean to moderately high levels; and the curves for teachers appeared to occupy a slightly narrower part of the latent trait compared to parents. The same findings were reproduced with the use of the Disruptive Behaviour Rating Scale (DBRS) in Gomez et al. [6]. Li et al. [8] using a different sample found that the information was centred around the mean using cases and controls using a different set of psychometric tools. However, to our knowledge, no other study has examined the effects of different informant aggregation algorithms with regards to diagnostic stability.

A range of studies have found that diagnostic labelling and the distribution of ADHD subtypes varies according to the diagnostic aggregation algorithm used [9, 10]. There are three different aggregation algorithms proposed to integrate parent-teacher information: (i) ‘average-rule’, (ii) ‘or-rule’ and (iii) ‘and-rule’ approaches (detailed description in Martel, et al. [11]). Briefly, the ‘or-rule’, based on the Diagnostic and Statistical Manual-IV (DSM-IV) [12] field trials, defines a symptom as present if either the parent or the teacher endorses that symptom. In other words, parents and teachers can independently endorse any one symptom without agreement on that specific symptom so the final total symptom count could be based on a different set of symptoms from each informant. In contrast, the ‘and-rule’ requires the same ADHD symptoms to be present both at home and at school, therefore both parents and teachers must agree on the presence of each symptom, so the total score is based on the same set of symptoms. The ‘average-rule’ considers symptom ratings as a probabilistic function of ADHD severity and can be averaged across raters to reduce the effects of rater biases, in particular when they disagree. Diagnostic rates are lower when using the ‘and-rule’ compared to the ‘or-rule’.

One of our recent studies, with the same sample, examined measurement invariance and established that parent and teacher informant assessments (INFAs) essentially rate different kinds of behaviours expressed in different settings, instead of their differences arising from measurement bias [13]. Lack of measurement invariance (or presence of *differential item functioning* in the IRT framework) exists when an item functions differently based on group membership: that is, when individuals with the same trait levels have different probabilities of endorsing the symptom criterion based only on group membership. Accordingly, we investigated whether for the same underlying levels or the traits (Inattention (IA) or Hyperactivity/impulsivity (HI) separately) parents and teachers have the same probability of endorsing a symptom criterion; and the result was negative. Teacher-INFAs were fundamentally different from that of parents, which we argued, could be attributable to different behaviours at home and at school, to different understanding of the symptom criteria by teachers and parents, or most likely to a combination of different child behaviour and different rater understanding—a view in keeping with those proposed by Willcutt et al. [14], Hartman et al. [4] and Martel et al. [11].

Having established the lack of measurement invariance between parent and teacher-INFAs, we considered important to explore next the patterns in parents and teacher-INFAs across the latent trait continuum /symptom dimension using an item-response theory (IRT) framework.

Previously, Garcia-Rosales et al. [15] explored the parent-INFAs in ADHD probands and their unaffected siblings using IRT. Item characteristic curves (ICC) of the symptom criteria of IA or HI were found to spread across each trait continuum (different difficulty/severity parameters) with varying discrimination abilities. The authors inferred this result as showing that all criteria are useful in measuring the traits, as ICCs of different items span over different levels of the continuum.

To make an ADHD diagnosis, DSM-5 and ICD systems require the presence of symptoms pervasive across settings. In real-life clinical diagnostic evaluation, clinicians therefore need to obtain valid and reliable information from informants more than one setting in a cost-effective manner. Apart from mental state examination of the child and a qualitative school report from the teacher, a clinician usually relies on additional information from two key sources: (i) clinical interviews of parents; and (ii) rating scale information from teachers. However, how to best utilize the different pieces of information and how to best combine them has not been examined in depth using IRT, in particular, using the ‘and-rule’ and the ‘or-rule’.

In this study, we utilised IRT to explore (a) the IRT properties of the teacher-INFAs for each symptom (severity, discrimination, information) across the symptom dimension (b) the IRT properties of the aggregated ratings (‘or-rule’ and ‘and-rule’), and (c) the differences in total symptoms endorsed by parent and teacher INFAs and the implications of the aggregation methods in diagnosis. The statistical robustness of this study is underpinned by the previous one [13] where a formal comparison between parent and teacher INFAs was carried out.

## Methods

### Sample

#### Recruitment

The International Multicentre AD/HD Genetics (IMAGE) project was an international collaborative study funded by the National Institute of Mental Health (NIMH) to identify candidate genes, genetic markers, and quantitative trait loci associated with ADHD. The sample consisted of European Caucasian subjects recruited from twelve specialist host centres in eight countries: Belgium, Germany, Holland, Ireland, Israel, Spain, Switzerland and the United Kingdom. Ethical approval for the study was obtained from Ethical Review Boards within each country and informed consent obtained from the children and their families. All children (probands and siblings) were aged 5 to 17, had an IQ ≥ 70, were of European Caucasian descent, and had access to at least one biological parent for DNA collection. Exclusion criteria applying to both probands and siblings included autism, epilepsy, IQ < 70, brain disorders, and any genetic or medical disorder associated with externalizing behaviours that might mimic ADHD. A detailed description of the study design and diagnostic approach has been published elsewhere [16, 17].

#### Sample for the present analyses

The initial sample from the IMAGE project consisted of 3229 entries, of which 1788 were selected with complete Parental Account of Children’s Symptoms (PACS) data and ADHD ratings by teachers. Among those, 1383 children were initially referred with an ADHD diagnosis (as ‘probands’) and 405 were siblings of the probands (detailed description found in Garcia-Rosales et al. [15]). For the present analyses, the data of one child per family were used to ensure independent observations. Siblings were prioritised to preserve a wider range of symptom levels. In cases of multiple siblings, one sibling was randomly selected. As a result, the current sample consists of 1017 (73.5%) children initially recruited as ‘probands’ in the IMAGE project and 366 (25.5%) initially recruited as ‘siblings’. Among the 366 children initially recruited as ‘siblings’, 243 (66.4%) also received an ADHD diagnosis after recruitment despite not initially being referred as probands. In the final sample there were 247 females (17.9%) and 1136 males, aged from 4 to 19 years (*M* = 10.9, SD = 2.9 years). The two genders^1^ did not differ with respect to age (*t*(1358) = 1.13, *p* = 0.261).

### ADHD symptom measures

#### Conners’ rating scale

The Long Version of the Conners’ Teacher Rating Scale (CTRS-R:L) [18] was used to obtain teacher ratings of the 18 symptoms of ADHD from the DSM-IV. Items from the ‘L’ (inattentive (IA) 9 items) and ‘M’ (hyperactive-impulsive (HI) 9 items) subscales correspond with the 18 DSM-IV [19] school criteria. Each item on the Conners’ scales was rated on a four-point Likert scale; 0 = not true at all (never, Seldom); 1 = Just a little true (Occasionally); 2 = Pretty much true (Often, Quite a bit); 3 = Very much true (Very often, Very frequently). Each of the 18 DSM school items was scored as present if the Conners item was scored positively, i.e. 0/1 designated as negative and 2/3 as positive. This dichotomic scoring is a more practical approach to clinical reality.

#### Parental account of childhood symptoms (PACS)

ADHD symptoms derived from parental accounts were obtained from PACS ratings. PACS is a standardized semi-structured, investigator-based interview, developed as an instrument to provide an objective measure of a child’s behaviour. For full details, see sister paper by Garcia-Rosales et al. [15]

The choice of different scales to collect information from parents and teachers should constitute a closer reflection of clinical reality where clinicians collect a lot more detailed and historical information in the home setting from parents usually present in the clinic as well as information from teacher observations over the course of months sometimes years encapsulated in a questionnaire as opposed to a formal clinical interview.

The abbreviations of DSM items used in this report are adapted from those used in the DSM-IV field trial [20] and are listed in the online supplementary materials (Tables S1 and S2).

### Data analyses

Teacher-INFAs and combination ratings were examined using IRT (see glossary of IRT terms in Table S3). According to IRT, a latent trait (*θ*) may not be directly measurable but it can be measured indirectly in a multivariate manner via a set of observed items. Here we implemented the two-parameter logistic IRT model [21], where the probability of a symptom being endorsed is modelled as a function of the trait and the specific item’s characteristics denoted by two parameters: difficulty/severity and discrimination. For further details on the 2-PL IRT model within ADHD, see Garcia-Rosales et al. [15]. Mplus software was used for the IRT analysis [22] All other statistical analyses were carried out using R [23, 24].

Based on the ***group invariance property*** of the IRT item parameters, (see reference [21], chapter 3), item parameters are a property of the items themselves, and not the group responding to the item. This has the direct result that since our sample consists of high ability individuals (individuals with high IA and HI traits) our results depict the upper tail of the item characteristic curves (ICCs) of the items. The parameters are not expected to be different to those of the general population according to the IRT model. That is, to use Baker’s words, the IRT estimated parameters are expected to be “in the same ballpark” to the ones that would have emerged, had our sample been a random sample rather than from the general population.

For the agreement between different parent and teacher-INFAs, we used the percentage of agreement between informants and the intraclass correlation coefficient [25] was used for the total scores. Logistic regression models were used to estimate the odds of an item being endorsed by either INFA, as predicted by gender, adjusting for age. We have also added the same results adjusted for the general score of ADHD symptoms.

## Results

Our sample overlaps with the Garcia-Rosales et al. [15] analysis by over 90% and therefore the parental ICCs are as expected almost identical to those presented there. For accuracy reasons, all ICCs are presented for parent and teacher-INFAs estimated using this exact sample (see Tables S1 and S2). Please note that Vitoratou & Garcia-Rosales et al. [13] present a formal statistical comparison of the intercepts and slopes (thresholds and loadings) related to the IA and HI models and the effect of gender and age per informant. Here, we study differences using the IRT item information curves (IICs).

### Psychometric properties of the teacher-INFAs using the IRT model: severity, discrimination and information

**Fig. 1:**
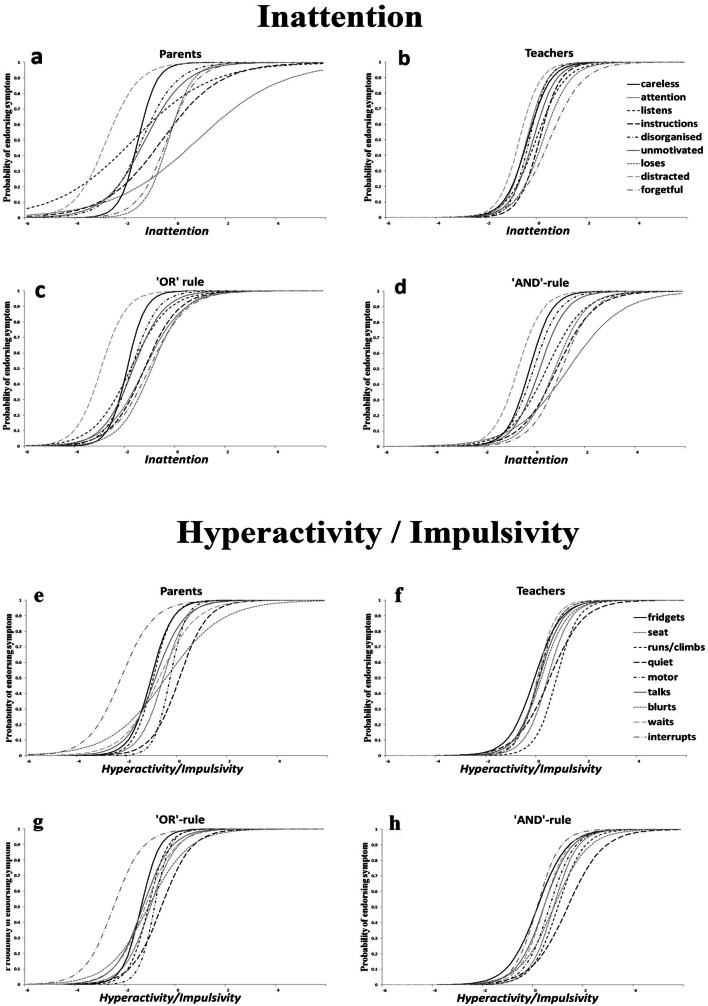
Item characteristic curves per informant assessment—IA and HI

In terms of IA (Fig. 1b), *distracted* was the least severe symptom (meaning it was endorsed even at low IA levels). Higher levels of IA were required for *loses* and *forgetful* to be endorsed (located at the upper end of the trait continuum, thus endorsed for children with higher levels of IA). The slopes of all ICCs were broadly similarly steep (especially in comparison with the parental curve); even small differences in the levels of IA (*x*-axis) corresponded to large differences in the probability of endorsing this symptom (*y*-axis), and this applied to all IA symptom criteria.

Regarding HI (Fig. 1f), *fidgets* was the least severe item contrasting with *runs/climbs* being the most severe. That is, teachers would endorse *fidgets* even for children with HI levels at the lower end of the continuum, however would require high levels of HI to endorse *runs/climbs*. *Quiet* was the least discriminating item and *motor* was the most discriminating symptom. That is, endorsement of *quiet* discriminated less between children with and without HI trait levels, compared to the endorsement of *motor*.

In both IA and HI, we observe that the ICCs are clustered together, close to the average value (0) and with broadly similar slopes, in contrast to the parent-INFAs.

**Fig. 2:**
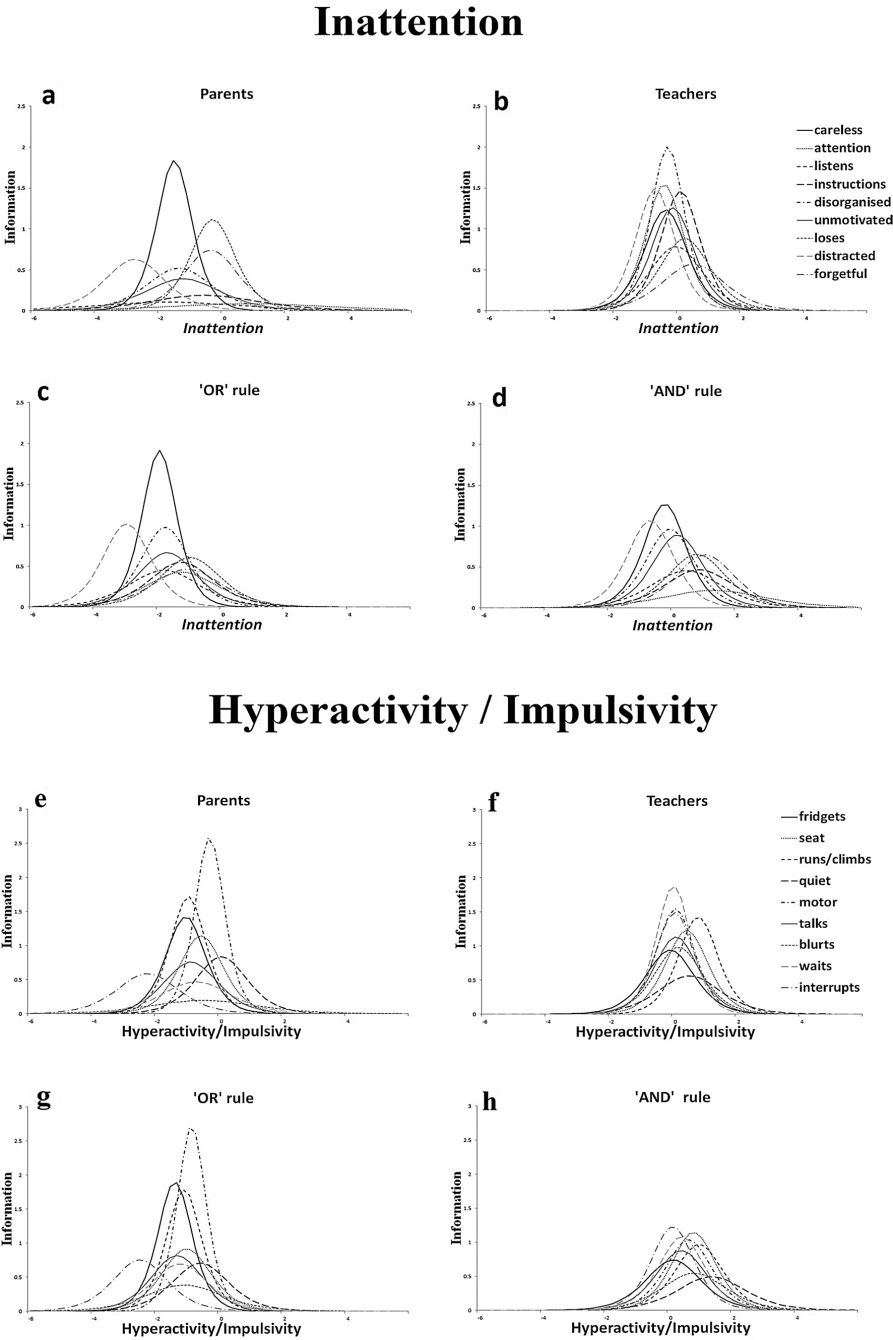
Item information curves per informant assessment—IA and HI

The item information curves (IIC), which correspond to the teacher-INFAs are presented in Fig. 2, separately for IA and HI. Regarding IA (Fig. 2b), the most informative (precise, reliable) item was *disorganised* and the least one was *forgetful*. For HI (Fig. 2f), the most informative item was *waits* and the least one was *quiet*. However, in both IA and HI measurements, most of the items are somewhat equivalent in terms of precision with a peak close to the average trait levels (plus-minus one standard deviation; SD). That is, the teacher-INFAs in all symptoms are mostly reliable for the average person, in terms of IA/HI levels. In contrast to the parental curves, there were no symptoms isolated in terms of precision at a certain point on the continuum (as is the case in parent-INFAs where *distracted*, for instance, peaks at 3 SDs below the average IA).

See Tables S1 and S2 for parameter estimates for both parent and teacher-INFAs and Figures S1 and S2 for the corresponding ICCs and Figures S2 and S6 for all IICs for each gender.

**Fig. 3:**
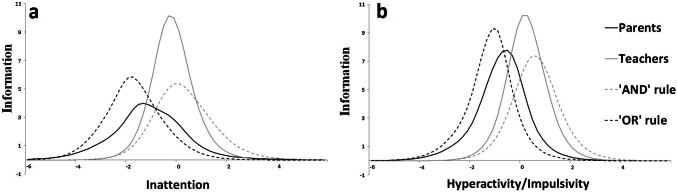
Total information curves per informant assessment—IA and HI

Figure 3 presents the total information curve per INFA. On one hand, teacher-INFAs appear to be the most reliable, located plus-minus two standard deviations around the average symptom dimension (IA and HI). On the other, on lower levels of the continuum, parent-INFAs demonstrate higher reliability. That is, for children with lower ADHD traits than average, the parent-INFAs are more reliable.

### Psychometric properties of the combined ratings using the IRT model: severity, discrimination and information per aggregation method

The ‘or-rule’ ICCs occupied a similar location in the continuum as those of the parents’ ICCs but they were ‘compressed’ closer together than those of the parents’ (see Fig. 1c, g). In other words, their severity was mostly influenced by the parent ratings whilst their discrimination ability was influenced by the teacher ratings. In contrast, the ‘and-rule’ ICCs were closer in location to that of the teachers’ ICCs but rendered the ICCs more spread out (Fig. 1d, h). Therefore, in terms of severity and discrimination, the influence of parent and teacher-INFAs on the ‘and-rule’ items is reversed compared to that of the ‘or-rule’ items*.* Patterns were broadly similar for IA and HI dimensions as well as the precision of the items, depicted in the IICs (Fig. 2).

Figure 3 presents the total information curve (TIC) per aggregation method. The ‘and-rule’ ratings appear to be more reliable over the range between +/− 2 two standard deviations around the average on both symptom dimensions (IA and HI). On lower levels on the continuum, ‘or-rule’ ratings demonstrate higher reliability. That is, for children with lower ADHD traits than average, the ‘or-rule’ information is more reliable, influenced by the parent-INFAs.

### Differences in total symptoms endorsed by parent and teacher INFAs and the implications of the aggregation methods in diagnosis

To examine gender differences in specific symptoms we used logistic regression: that is, modelling each item (i.e. presence or absence) as the dependent variable predicted by gender, adjusted for age, separately for parents and teachers and for IA and HI. The Benjamini and Hochberg [26] correction for multiple comparisons was used, on reviewer’s recommendation. The odds of endorsing a particular symptom were higher in boys than girls of the same age, according to teachers-INFAs. The only exception was in *forgetful* where boys and girls of the same age had equal odds to be considered as manifesting the symptom. Whilst the odds were higher for boys in the parents’ ratings as well, in most IA symptom the differences in the odds due to gender were not significant. With regards to the HI symptoms (Table 1), according to parents-INFAs, the odds of endorsing a particular HI symptom were higher in boys than girls of the same age, apart from *talks*.

**Table 1:** Odds Ratios of symptom considered present by parent and teacher informant assessments in relation to gender (reference level = females—adjusted for age)

Inattention			Hyperactivity/Impulsivity		
Symptom	Parents	Teachers	Symptom	Parents	Teachers
Careless	2.4**	2.0**	Fidgets	2.4**	2.8**
Attention	^+^1.4*	1.7**	Seat	1.5**	3.6**
Listen	1.5*	2.0**	Runs/climbs	3.0**	3.6**
Instructions	1.5*	1.9**	Quiet	1.5**	2.4**
Disorganised	1.9**	1.8**	Motor	1.6**	2.8**
Unmotivated	2.2**	2.1**	Talks	^+^1.3	1.7**
Loses	^+^1.2	1.8**	Blurts	1.5**	2.1**
Distracted	^+^1.5	2.3**	Wait	2.1**	2.9**
Forgetful	^+^1.3*	^+^1.1	Interrupts	2.1**	2.4**

*p < 0.05; **p < 0.001; ^+^non significant after adjusting for multiple comparisons (Benjamini–Hochberg adjustment)

Parents endorsed significantly more symptoms (Mann–Whitney test for IA: median 7 vs 6 symptoms, *p* < 0.001; for HI: median 7 vs 4 symptoms, *p* < 0.001) than teachers. The percentage of children rated as having a symptom by the parents-only ranged from 12 to 56%. On the other hand, the percentage of children rated by the teachers-only was comparatively lower with a range of 2 to 19% (with the exception of *attention* at 39%). The agreement ranged from 41% for runs/climbs to 65% for careless and 74% for distracted, with most of the symptoms having about 50% agreement between raters. On the sum-score level, the intraclass correlation coefficients were low for both IA and HI (*r* = *0*.17 and 0.12 respectively) indicating very low agreement.

These results have direct implications on the aggregation criteria and therefore on diagnosis. Table 2 shows the number and proportions of children who met the threshold criterion of the 6-item cut-off for each dimension as defined by parent and teachers-INFAs alone, and as defined by the ‘and’ versus ‘or rules’ of aggregation. As described earlier, the ‘and-rule’ aggregation requires both parent and teacher-INFAs to consider the same symptom present; while the ‘or-rule’ aggregation allows either parent or teacher-INFAs to consider a symptom present. Compared to teachers, the parents rated 27% more children meeting the 6-symptom threshold for the IA domain and 33% for the HI domain. As we have established in a paper by the same authors, with the same sample, this discrepancy stems from the fact different INFAs are capturing different aspects of children’s behaviour. Parents and teachers agreed on only 30% of the ‘IA cases’ and on 23% of the ‘HI cases’. The frequency of the potential ‘cases’ (i.e. those meeting the 6-symptoms-present criterion) using the ‘or-rule’ almost tripled that of the ‘and-rule’ as shown in Table 2.

**Table 2:** Number of children with six symptoms present according to informant assessment (INFA)

INFA	Inattention	Hyperactivity
	*N* (%)	*N* (%)
Parents	1034 (75%)	981 (71%)
Teachers	732 (53%)	521 (38%)
OR-rule	1254 (91%)	1156 (84%)
AND-rule	419 (30%)	316 (23%)

## Discussion

Consistent research findings indicate that parents and teachers observe genuinely different ADHD behaviours with unique aspects of expression in their respective setting. Yet to date, there is a gap in the literature in exploring using IRT, how to best combine the information. To make a clinical diagnosis, a clinician routinely utilises interview information from the parents and rating scale information from the teachers; the extent to which diagnostic stability is influenced by the aggregation algorithms has not been examined in detail. Our study utilized IRT and regression analysis to describe and evaluate the utility of teacher information as well as the implications of using different aggregation algorithms to combine teacher and parent information, based on the ‘and-rule’ versus the ‘or-rule’.

There were four key findings in this study. Firstly, all teacher-INFAs demonstrated similar levels of precision for all symptoms as appraised by teachers, in both dimensions. With respect to severity and discrimination, the ICCs had similar slopes and were relatively close to one another, indicating that the teachers were locating the part of the continuum associated with the presence of most symptoms and thus atypical behaviours. These observations were not replicated in parent ratings with the same children [15]. Compared to Gomez [5], our findings concur with the high alpha values in teacher ratings for *careless* and *attention* and a low value for *forgetful*, however our results do not concur for the alpha values in the HI dimension. In our study teacher curves were very close to one another around the mean similarly to Li et al. [8]. In Gomez [5] and Gomez et al. [6], most information was located in moderate to high trait levels and are slightly more spread out. Similarly to Gomez [6], teacher-INFAs alpha values were high for *careless*, *attention* and *instructions,* low for *quiet* and high for *motor*. Arias et al. [7] found that the most discriminative items were *distracted*, *instructions*, *leaves seat*, *runs/climbs* for which we have found high alpha values. The differences observed may be attributable to the differences in samples as well as the different psychometric tools used. Secondly, when either parent or teacher endorsement was considered enough for the presence of the symptoms, the resulting ICC curves were located to the left of the trait continuum. That is, the symptoms were endorsed in the presence of low trait levels, indicating the influence of the parental endorsement. However, the slopes of the curves indicate that the influence of the teacher-INFAs added to the discriminative power of the items. When the endorsement of both informants was required to consider a symptom present (i.e. ‘and-rule’), the ICCs were located closer to that of the teacher-INFA ICCs, but made the parent-INFA more spread out (Fig. 1d, h). Thirdly, with regard to the symptoms endorsed by parents and teachers, our results replicated previous research reporting low parent-teacher agreement (ranging from 0.09 to 0.43). Teachers rated higher symptoms in boys than girls, and parental information was less influenced by gender. Fourth, the proportions of children meeting the six-item threshold for both IA and HI subdomains as defined by teacher-INFA were lower; and such proportions also varied widely according to the algorithm used to aggregate teachers’ ratings (‘and-rule’ versus ‘or-rule’).

It is likely that a more accurate diagnosis (specific) could be made based on teacher-INFA as they provide richer information within a narrower middle window. Parent-INFA described the symptoms along the continuum of the traits as the ICC were spread from very low to very high trait levels; in contrast, for teachers, symptoms are clustered within that narrower middle window. As the number of symptoms is one of the criteria for the diagnosis of ADHD, this middle window potentially constitutes the diagnostic threshold where cases could be differentiated from non-cases. Teacher information would therefore be more likely helpful in informing diagnosis, whereas parental information would help gathering information across the full continuum. These findings have a bearing in terms of re-thinking the diagnostic criteria for ADHD. Consistent with our view, Gomez [5] noted that the information from teachers increases significantly, when the rating becomes higher.

Finally the ‘potential case status’ (defined here solely by the 6-item threshold) varied widely depending both on the source of information and on the aggregation algorithm employed (‘and-rule’ versus ‘or-rule’). Our findings are in line with previous studies which focused on caseness and subtype stability across aggregation methods in both clinical [10, 25] and community samples [9]. In our study, parents tended to report more symptoms i.e. the proportions of children meeting the six-item threshold for both IA and HI subdomains as defined by parental information were higher; furthermore, using the ‘or-rule’ algorithm to combine teacher ratings yielded even higher proportions of children reaching this threshold. In contrast, the ‘and-rule’, which required symptom confluence by definition, yielded much lower rates.

Several limitations need to be considered. Different methodological tools were used to assess ADHD symptoms in our study: PACS interviews for parents and Conners rating scales for teachers. PACS provides far more detailed and minute information compared to Conners. This is in keeping with clinical practice where clinicians often have more information from parents; but often are sent a completed ADHD questionnaire such as Conners by teachers. For this reason, our results should be closer to the busy clinician’s everyday practice. Our sample consisted of ADHD cases ascertained from psychiatric and paediatric clinics with their non-ADHD siblings forming the comparison group, so the comparison group was not independently ascertained. Due to genetic relatedness, there were likely many subthreshold ADHD cases among the siblings, which probably reduced our item discrimination parameters. Furthermore, the sample analysed consisted of a high proportion of probands. Despite this, due to the *group invariance property* of the IRT item parameters, our findings are potentially statistically generalizable to the general population, however other factors need to be taken into account such as the effects of instruments and measurement factors which could not be fully disentangled from that of raters. Our findings must be regarded as preliminary before replication using either community samples or clinic samples with independently ascertained controls. Our sample also consisted of participants recruited from different centres located in different European countries, Müller et al. [17] have demonstrated the multi-level nature of our data with potential factors influencing symptom levels as well as age and gender effects across centres and countries inherent in most multicentre studies. It would also be helpful to ascertain the effects of ethnicity and socioeconomic status on the symptoms on one hand and on the other look into individual classrooms. Given the cross-sectional design of the IMAGE study, the effects of developmental trajectories could not be modelled. Our findings must therefore be considered as preliminary and should be interpreted with some caution and needing further replication with regard to the above-mentioned limitations.

## Conclusions and Clinical Implications

Our findings identify the specific merits of the ‘and-rule*’* and ‘or-rule’ with a finer differentiation on information contribution and utility, such as determining severity and diagnosis of ADHD in lower-level severity groups. Parents are better at screening than diagnosing. Overall, the ‘or-rule’ appears more appropriate for capturing cases with milder and more nuanced symptom expression (more sensitive); and the ‘and-rule’ for determining and indexing severity in the clinical range (more specific). Therefore, for diagnosing ADHD in under-identified groups (i.e. older, female and especially younger females), the ‘or-rule’ may be more applicable. The DSM-5 [27] has introduced a specifier for ‘current severity’ (from ‘Mild’, ‘Moderate’ to ‘Severe’) with levels defined by both number of symptoms and severity of impairment. For earmarking severity, the ‘and-rule’ appears more accurate, instead of symptom count (as proposed by DSM-5) due to a potential ceiling effect of the total count yielded by the ‘or-rule’.

Overall, our findings highlight important and valuable aspects of different INFAs and their combinations. Each group uniquely provides clinically relevant and valuable information.

## Electronic supplementary material

Below is the link to the electronic supplementary material.

Supplementary file1 (DOCX 1405 kb)

## Abbreviations

ADHD: Attention Deficit/Hyperactivity Disorder
DSM-IV: Diagnostic and statistical manual-IV
IRT: Item-response theory
IA: Inattention
HI: Hyperactivity/impulsivity
INFA: Informant assessment

## Footnotes

1 Studies vary in whether they record sex or gender. We have chosen to record the distinction between male and female as “gender”. At the time the data was collected, there was little awareness of gender identity issues.

## References

[1] National Institute of Health and Care Excellence (NICE). (2018) Attention Deficit Hyperactivity Disorder: diagnosis and management. NICE guideline. www.nice.org.uk/guidance/ng87 29634174

[2] Narad ME, Garner AA, Peugh JL, Tamm L, Antonini TN, Kingery KM, Simon JO, Epstein JN (2015). Parent–teacher agreement on ADHD symptoms across development. Psychol Assess.

[3] Tripp G, Schaughency EA, Clarke B (2006). Parent and teacher rating scales in the evaluation of attention-deficit hyperactivity disorder: contribution to diagnosis and differential diagnosis in clinically referred children. J Dev Behav Pediatr.

[4] Hartman CA, Rhee SH, Willcutt EG, Pennington BF (2007). Modeling rater disagreement for ADHD: are parents or teachers biased. J Abnorm Child Psychol.

[5] Gomez R (2008). Item response theory analyses of the parent and teacher ratings of the DSM-IV ADHD rating scale. J Abnorm Child Psychol.

[6] Gomez R, Vance A, Gomez A (2011). Item response theory analyses of parent and teacher ratings of the ADHD symptoms for recoded dichotomous scores. J Atten Disord.

[7] Arias VB, Nuñez DE, Martínez-Molina A, Ponce FP, Arias B (2016). Hierarchy and psychometric properties of ADHD symptoms in Spanish children: an application of the graded response model. PLoS ONE.

[8] Li JJ, Reise SP, Chronis-Tuscano A, Mikami AY, Lee SS (2016). Item response theory analysis of ADHD symptoms in children with and without ADHD. Assessment.

[9] Rowland AS, Skipper B, Rabiner DL, Umbach DM, Stallone L, Campbell RA, Hough RL, Naffel AJ, Sandler DP (2008). The shifting subtypes of ADHD: classification depends on how symptom reports are combined. J Abnorm Child Psychol.

[10] Valo S, Tannock R (2010). Diagnostic instability of DSM–IV ADHD subtypes: Effects of informant source, instrumentation, and methods for combining symptom reports. J Clin Child Adolescen Psychol.

[11] Martel MM, Schimmack U, Nikolas M, Nigg JT (2015). Integration of symptom ratings from multiple informants in ADHD diagnosis: a psychometric model with clinical utility. Psychol Assess.

[12] American Psychiatric Association (2000). Diagnostic and statistical manual of mental disorders.

[13] Vitoratou S, Garcia-Rosales A, Banaschewski T, Sonuga-Barke E, Buitelaar J, Oades RD, Rothenberger A, Steinhausen H-C, Taylor E, Faraone SV, Chen W (2019). Is the endorsement of the Attention Deficit Hyperactivity Disorder symptom criteria ratings influenced by informant assessment, gender, age, and co-occurring disorders? A measurement invariance study. Int J Methods Psychiatr Res.

[14] Willcutt EG, Carlson CL (2005). The diagnostic validity of attention-deficit/hyperactivity disorder. Clin Neurosci Res.

[15] Garcia-Rosales A, Vitoratou S, Banaschewski T, Asherson P, Buitelaar J, Oades RD, Rothenberger A, Steinhausen H-C, Faraone SV, Chen W (2015). Are all the 18 DSM-IV and DSM-5 criteria equally useful for diagnosing ADHD and predicting comorbid conduct problems?. Eur Child Adolesc Psychiatry.

[16] Chen W, Zhou K, Sham P, Franke B, Kuntsi J, Campbell D, Fleischman K, Knight J, Andreou P, Arnold R, Altink M (2008). DSM-IV combined type ADHD shows familial association with sibling trait scores: a sampling strategy for QTL linkage. Am J Med Genet Part B.

[17] Müller UC, Asherson P, Banaschewski T, Buitelaar JK, Ebstein RP, Eisenberg J, Gill M, Manor I, Miranda A, Oades RD, Roeyers H, Rothenberger A, Sergeant JA, Sonuga-Barke EJ, Thompson M, Faraone SV, Steinhausen HC (2011). The impact of study design and diagnostic approach in a large multi-centre ADHD study. Part 1: ADHD symptom patterns. BMC Psychiatry.

[18] Conners CK, Sitarenios G, Parker JD, Epstein JN (1998). Revision and restandardization of the Conners Teacher Rating Scale (CTRS-R): factor structure, reliability, and criterion validity. J Abnor Child Psychol.

[19] American Psychiatric Association (2000). Diagnostic and statistical manual of mental disorders: DSM-IV-TR.

[20] Frick PJ, Lahey BB, Applegate B, Kerdyck L, Ollendick T, Hynd GW, Garfinkel B, Greenhill L, Biederman J, Barkley RA, McBURNETT KEITH (1994). DSM-IV field trials for the disruptive behavior disorders: symptom utility estimates. J Am Acad Child Adolesc Psychiatry.

[21] Baker F (2001). The basics of item response theory. ERIC clearinghouse on assessment and evaluation.

[22] Muthén LK, Muthén BO, Mplus User’s Guide Team, R. C. (2013). R: a language and environment for statistical computing.

[23] Landis JR, Koch GG (1977). An application of hierarchical kappa-type statistics in the assessment of majority agreement among multiple observers. Biometrics.

[24] Team RC (2013). R: a language and environment for statistical computing.

[25] Shrout PE, Fleiss JL (1979). Intraclass correlations: uses in assessing rater reliability. Psychol Bull.

[26] Benjamini Y, Hochberg Y (1995). Controlling the false discovery rate: a practical and powerful approach to multiple testing. J R Stat Soc.

[27] American Psychiatric Association (2013). Diagnostic and statistical manual of mental disorders.

