# Supplementary material for: Differential utility of teacher and parent–teacher combined information in the assessment of Attention Deficit/Hyperactivity Disorder symptoms
Source: Eur Child Adolesc Psychiatry. 2020 Apr 3;30(1):143–53. doi: 10.1007/s00787-020-01509-4 (PMC7864845; doi:10.1007/s00787-020-01509-4)
Supplement: Supplementary file 1 — Supplementary file1 (DOCX 1405 kb) [file 787_2020_1509_MOESM1_ESM.docx]

Supplementary Materials

## Item abbreviations

The abbreviations of DSM items used in this report are adapted from those used in the DSM-IV field trial (Frick et al, 1994) and are listed in *italic* as the followings:

*Inattention items.* (1) “Often fails to give close attention to details or makes careless mistakes in schoolwork, at work, or with other activities” (*Careless*). (2) “Often has trouble holding attention on tasks or play activities” (*Attention*). (3) “Often does not seem to listen when spoken to directly” (*Listen*). (4) “Often does not follow through on instructions and fails to finish schoolwork, chores, or duties in the workplace (e.g., loses focus, side-tracked)” (*Instructions*). (5) “Often has trouble organizing tasks and activities” (*Disorganised*). (6) “Often avoids, dislikes, or is reluctant to do tasks that require mental effort over a long period of time (such as schoolwork or homework)” (*Unmotivated*). (7) “Often loses things necessary for tasks and activities (e.g. school materials, pencils, books, tools, wallets, keys, paperwork, eyeglasses, mobile telephones)” (*Loses*). (8) “Is often easily distracted” (*Distracted*). (9) “Is often forgetful in daily activities” (*Forgetful*).

*Hyperactivity items*. “Often fidgets with or taps hands or feet, or squirms in seat” (*Fidgets*). “Often leaves seat in situations when remaining seated is expected” (*Seat*). “Often runs about or climbs in situations where it is not appropriate (adolescents or adults may be limited to feeling restless)” (*Runs/Climbs*). “Often unable to play or take part in leisure activities quietly” (*Quiet*). “Is often "on the go" acting as if "driven by a motor"’ (*Motor*). “Often talks excessively” (*Talks*). “Often blurts out an answer before a question has been completed” (*Blurts*). “Often has trouble waiting his/her turn” (*Wait*). “Often interrupts or intrudes on others (e.g., butts into conversations or games)” (*Interrupts*).

## Tables

##### Table S1: Item difficulty and discrimination parameters, per informant assessment - Inattention

| **IA symptom criteria** | **Difficulty parameters** | | | | **Discrimination parameters** | | | |
| --- | --- | --- | --- | --- | --- | --- | --- | --- |
|  | **parents** | **teachers** | **Or rule** | **And**  **rule** | **parents** | **teachers** | **Or rule** | **And**  **rule** |
| **Careless** | -1.6 | -0.5 | -2.0 | -0.3 | ***1.6*** | 1.3 | ***1.6*** | ***1.3*** |
| **Attention** | ***0.8*** | -0.5 | -1.3 | **1.3** | **0.3** | ***1.5*** | **0.8** | **0.6** |
| **Listen** | -1.8 | -0.1 | -1.9 | 0.4 | 0.4 | 1.1 | **0.8** | 0.8 |
| **Instructions** | -0.6 | *0.0 | -1.3 | 0.8 | 0.5 | 1.4 | 0.9 | 0.8 |
| **Disorganised** | -1.4 | -0.4 | -1.8 | -0.1 | 0.9 | 1.7 | 1.2 | 1.2 |
| **Unmotivated** | -1.3 | -0.2 | -1.8 | 0.1 | 0.7 | 1.3 | 1.0 | 1.1 |
| **Loses** | -0.4 | 0.2 | -1.0 | 0.7 | 1.2 | 1.2 | 0.9 | 1.0 |
| **Distracted** | **-2.8** | **-0.8** | **-3.0** | **-0.8** | 0.9 | 1.4 | 1.2 | 1.2 |
| **Forgetful** | -0.4 | ***0.4*** | ***-1.1*** | 1.0 | 1.0 | **0.9** | **0.8** | 1.0 |
| *Not statistically different than zero. Bold characters indicate the largest coefficient for the rater. Bold italic characters indicate the smallest coefficient within rater. | | | | | | | | |

##### Table S2: Item difficulty and discrimination parameters, per type of rating – Hyperactivity/Impulsivity

| **HI symptom criteria** | **Difficulty parameters** | | | | **Discrimination parameters** | | | |
| --- | --- | --- | --- | --- | --- | --- | --- | --- |
|  | **parents** | **teachers** | **Or rule** | **And**  **rule** | **parents** | **teachers** | **Or rule** | **And**  **rule** |
| **Fidgets** | -1.1 | **-0.2** | -1.5 | ***0.1** | 1.4 | 1.1 | 1.6 | 1.1 |
| **Seat** | -0.6 | 0.4 | -1.1 | 0.7 | 1.3 | 1.3 | 1.2 | ***1.3*** |
| **Runs/climbs** | -1.0 | ***0.7*** | -1.2 | 0.9 | 1.5 | 1.4 | 1.6 | 1.2 |
| **Quiet** | ****0.0*** | 0.5 | ***-0.7*** | ***1.3*** | 1.1 | **0.9** | 1.0 | **0.8** |
| **Motor** | -0.4 | *0.0 | -1.0 | 0.6 | ***1.9*** | 1.5 | ***2.0*** | 1.2 |
| **Talks** | -1.0 | *0.0 | -1.4 | 0.4 | 1.0 | 1.3 | 1.1 | 1.1 |
| **Blurts** | -0.5 | 0.1 | -1.2 | 0.7 | **0.5** | 1.2 | **0.7** | 0.9 |
| **Wait** | -0.8 | *-0.1 | -1.3 | 0.4 | 0.8 | ***1.6*** | 1.0 | 1.2 |
| **Interrupts** | **-2.3** | *0.0 | **-2.6** | ***0.1** | 0.9 | 1.4 | 1.0 | 1.3 |
| *Not statistically different from zero. Bold characters indicate the largest coefficient for the rater. Bold italic characters indicate the smallest coefficient within rater. | | | | | | | | |

##### Table S3. Glossary of IRT terms and concepts relevant to reported analyses

| **Term** | Meaning | Example |
| --- | --- | --- |
| **Item** | The question posed eliciting a categorical response | The 18 DSM-IV ADHD symptoms |
| **Latent trait** | Unobserved variable which is measured indirectly via the responses in the items (symptoms). | IA and HI (often referred to as symptom dimensions in psychiatric literature). |
| **Latent continuum** | The range of variation of the latent trait. | The range of values of IA and HI |
| **Item Response Theory** | Statistical model which relates the **latent trait** (psychiatrically: symptom dimension) to the probability of endorsing an **item** (psychiatrically: symptom). It provides us with the estimation of how severe a symptom is (**difficulty** parameter), whether it can differentiate between people at different points across the **latent continuum** (**discrimination** parameter), how precise an item is (**information**) and theory for how to combine information from several items | |
| **Item characteristic curve** | An S-shaped curve which depicts graphically the item characteristics (difficulty and discrimination) across the latent continuum. | The curve of the probability of endorsing *forgetful* across the continuum of IA. |
| **Difficulty parameter** | The difficulty parameter equals the trait levels of a person with 50% chance of being endorsed as exhibiting a symptom. It is related to the location of the ICC curve: more severe symptoms are placed further to the right and least severe symptoms are placed to the left. | The parent-INFA ICC of *distracted* is the one placed furthest to the left meaning that this item is the least severe among the symptoms of IA. In other words less of IA is required for this symptom to be present than for the other IA symptoms. |
| **Discrimination parameter** | The discrimination parameter is associated with the slope of ICC curve. A steep slope (large discrimination parameter) indicates that small increase in the latent trait amount(x-axis) corresponds to large increase in the probability of endorsing a particular symptom (y-axis). Therefore, the corresponding symptom can differentiate between people at different points around this trait value. | The symptom *careless* according to parental INFA is the one with the steepest ICC, meaning that it differentiates better than all other symptoms between people at different points across the IA continuum. |
| **Item Information Curve** | A bell shaped curve which depicts how informative (reliable, precise) a symptom is as an indicator of the latent trait across the continuum. A tall and narrow IIC indicates a symptom that is very precise but for a restricted part of the continuum, whilst a short and wide IIC indicates a symptom which is less precise but is informative for an extended part of the latent trait continuum. | The parent-INFAs IICs for *attention* and *listens* indicate that the former is informative in the middle of the IA continuum whilst the latter is mostly informative at the lowest end. |
| **Total characteristic or information curves** | A single curve which is the combined graphical representation of the complete information provided by a set of items for a particular latent trait (rather than one symptom at a time). | |


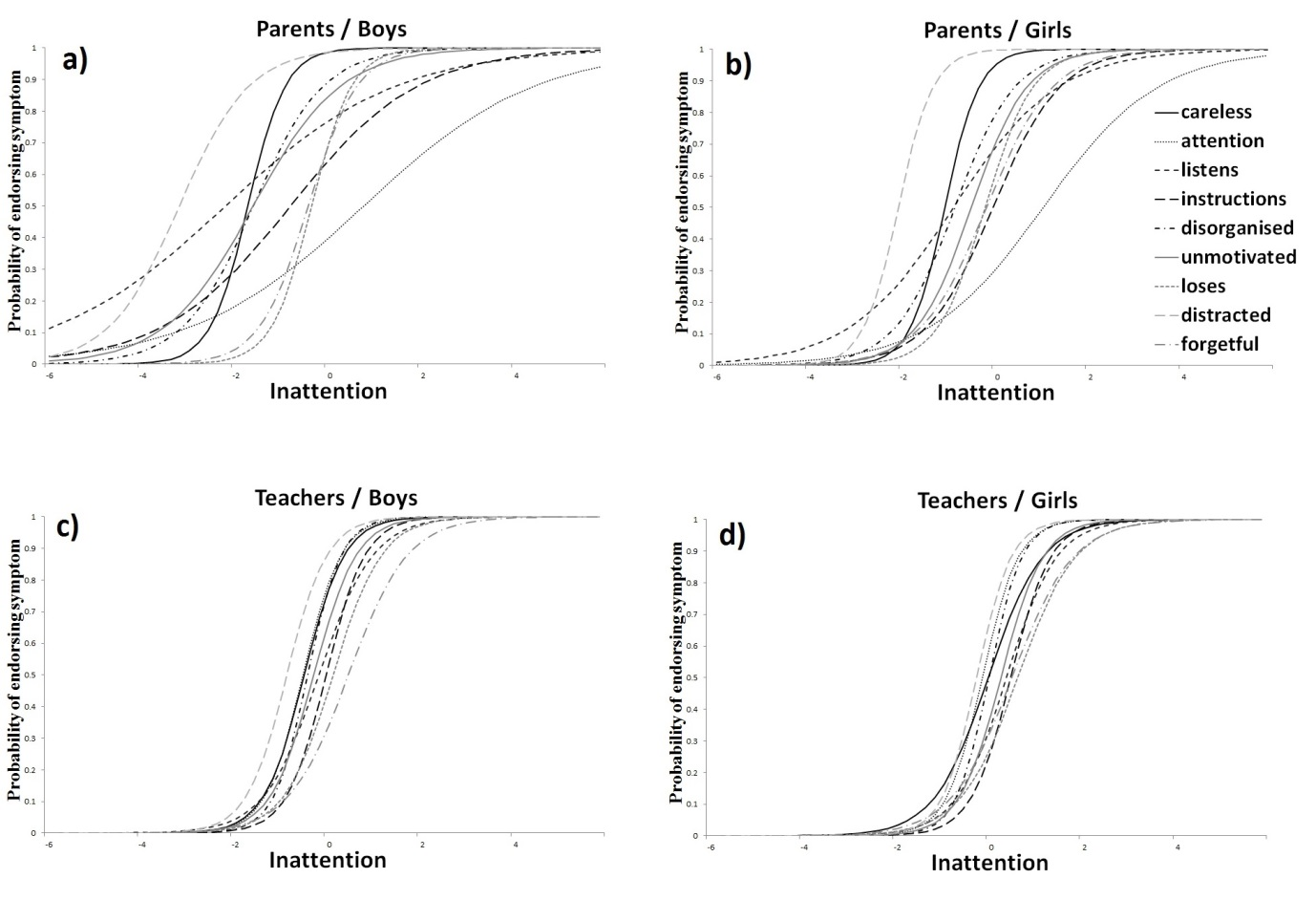


Figure S1 Item characteristic curves by gender - Inattention


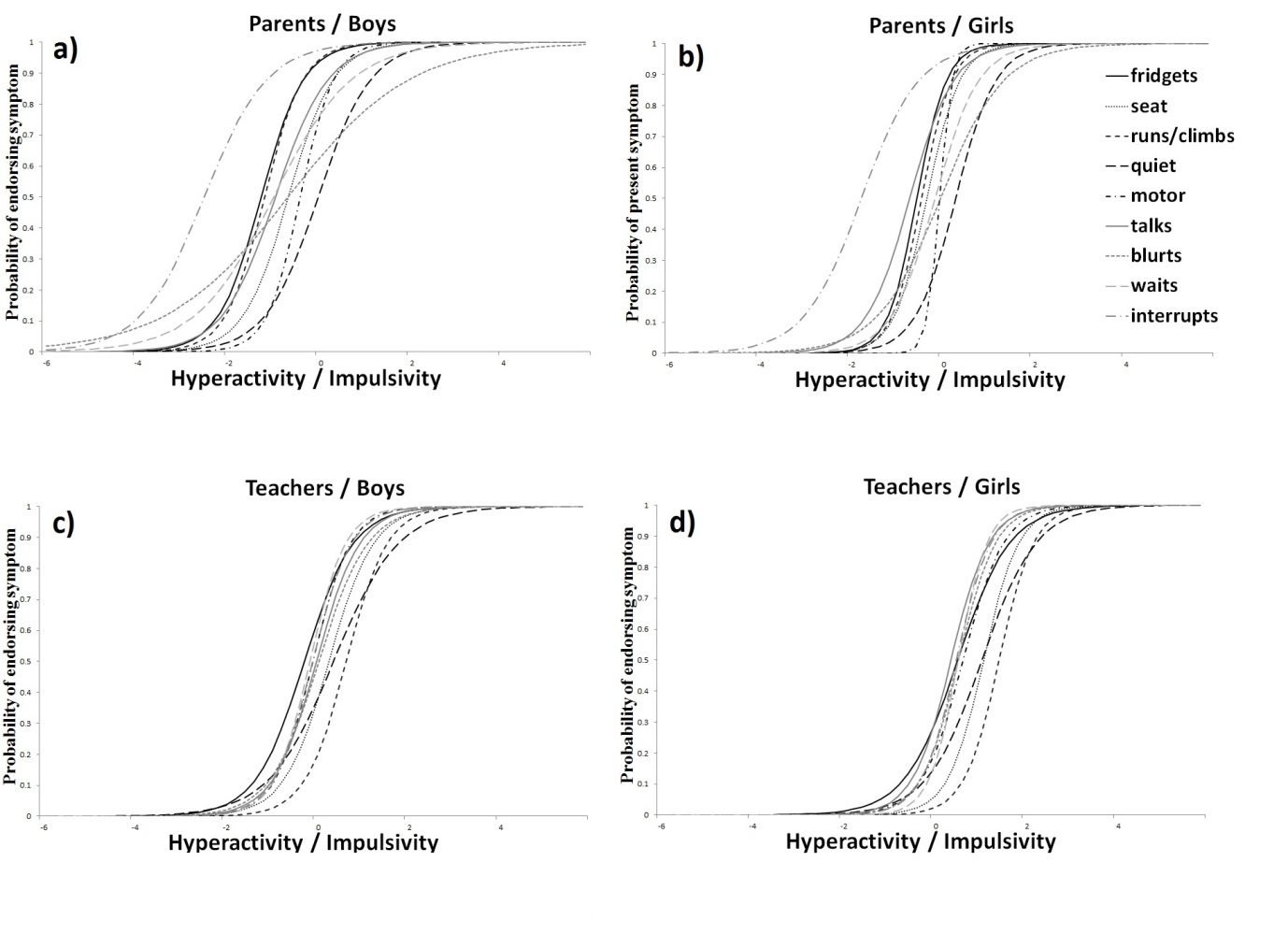


Figure S2: Item characteristic curves by gender – Hyperactivity / Impulsivity

**
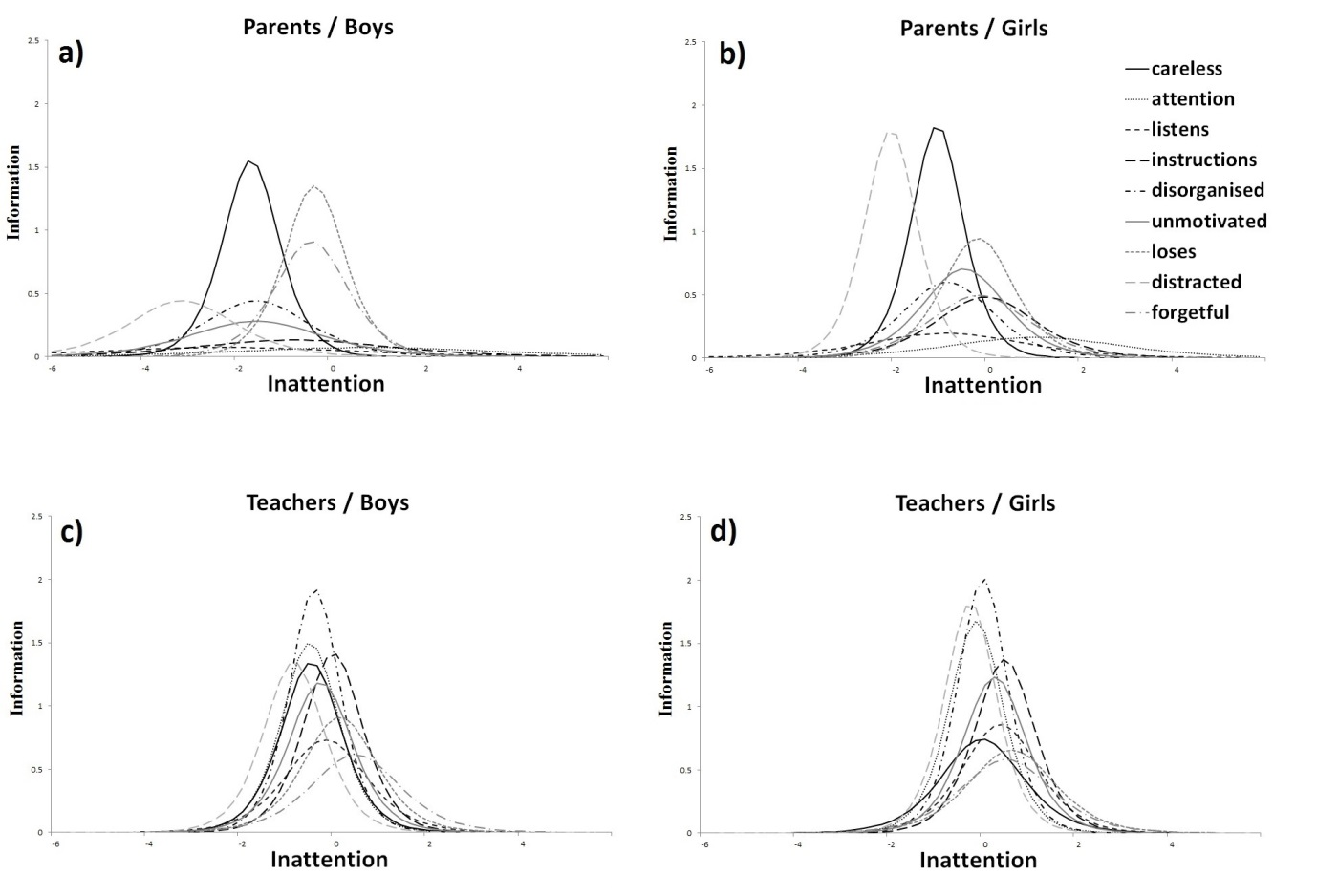
**

##### Figure S3: Item Information Curves by gender – Inattention


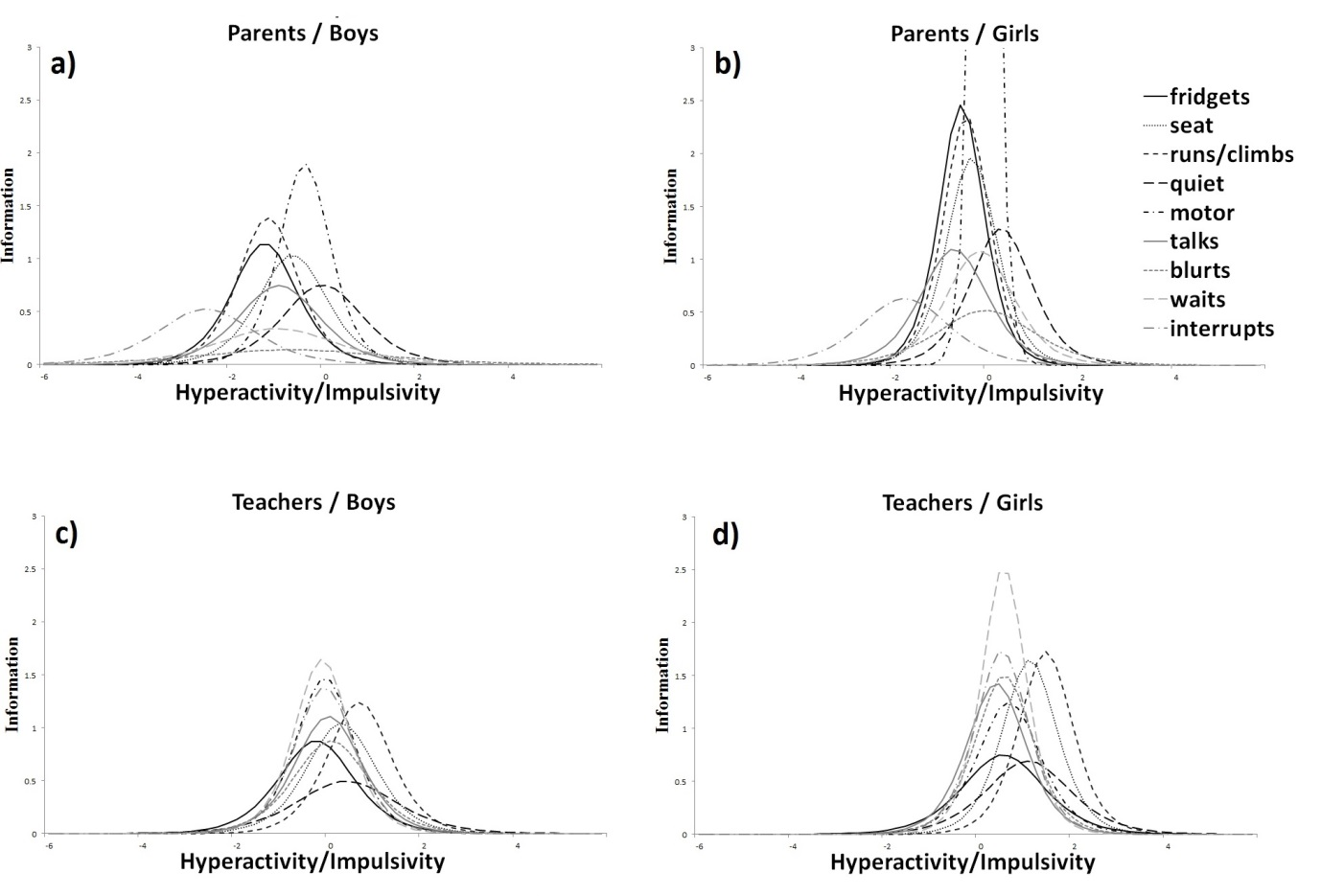


##### Figure S4: Item Information Curves by gender – Hyperactivity / Impulsivity
